# Antibody attributes that predict the neutralization and effector function of polyclonal responses to SARS-CoV-2

**DOI:** 10.1186/s12865-022-00480-w

**Published:** 2022-02-16

**Authors:** Harini Natarajan, Shiwei Xu, Andrew R. Crowley, Savannah E. Butler, Joshua A. Weiner, Evan M. Bloch, Kirsten Littlefield, Sarah E. Benner, Ruchee Shrestha, Olivia Ajayi, Wendy Wieland-Alter, David Sullivan, Shmuel Shoham, Thomas C. Quinn, Arturo Casadevall, Andrew Pekosz, Andrew D. Redd, Aaron A. R. Tobian, Ruth I. Connor, Peter F. Wright, Margaret E. Ackerman

**Affiliations:** 1 Department of Microbiology and Immunology, Geisel School of Medicine at Dartmouth, Dartmouth College, Hanover, NH USA; 2 Program in Quantitative Biological Sciences, Dartmouth College, Hanover, NH USA; 3 Thayer School of Engineering, Dartmouth College, 14 Engineering Drive, Hanover, NH 03755 USA; 4 Department of Pathology, Johns Hopkins School of Medicine, Baltimore, MD USA; 5 W. Harry Feinstone Department of Molecular Microbiology and Immunology, Johns Hopkins Bloomberg School of Public Health, Baltimore, MD USA; 6 Department of Pediatrics, Geisel School of Medicine at Dartmouth, Dartmouth-Hitchcock Medical Center, Lebanon, NH USA; 7 Department of Medicine, Division of Infectious Diseases, Johns Hopkins School of Medicine, Baltimore, MD USA; 8 Division of Intramural Research, National Institute of Allergy and Infectious Diseases, National Institutes of Health, Bethesda, MD USA

**Keywords:** SARS-CoV-2, IgG, IgM, IgA, Neutralization, Effector function

## Abstract

**Background:**

While antibodies can provide significant protection from SARS-CoV-2 infection and disease sequelae, the specific attributes of the humoral response that contribute to immunity are incompletely defined.

**Methods:**

We employ machine learning to relate characteristics of the polyclonal antibody response raised by natural infection to diverse antibody effector functions and neutralization potency with the goal of generating both accurate predictions of each activity based on antibody response profiles as well as insights into antibody mechanisms of action.

**Results:**

To this end, antibody-mediated phagocytosis, cytotoxicity, complement deposition, and neutralization were accurately predicted from biophysical antibody profiles in both discovery and validation cohorts. These models identified SARS-CoV-2-specific IgM as a key predictor of neutralization activity whose mechanistic relevance was supported experimentally by depletion.

**Conclusions:**

Validated models of how different aspects of the humoral response relate to antiviral antibody activities suggest desirable attributes to recapitulate by vaccination or other antibody-based interventions.

**Supplementary Information:**

The online version contains supplementary material available at 10.1186/s12865-022-00480-w.

## Background

The SARS-CoV-2 pandemic has resulted in over 300 million cases, 5.5 million deaths, and unprecedented social, economic, and educational impact despite interventions that have included quarantines, shutdowns, social distancing, and masking requirements. However, the pandemic has also led to international collaborations working toward understanding the disease and developing novel therapeutics and vaccines. To date, these efforts have resulted in several novel therapies and several vaccines approved for widespread deployment under emergency use authorization [1].

The success of these vaccines is thought to result in no small part to the potent antiviral activities of the antibodies they induce. While reinfections have been documented [2, 3], seropositivity and levels of neutralizing antibody are associated with highly reduced rates of re-infection [4–6], and passive transfer of plasma from convalescent donors has shown therapeutic efficacy in some studies [7–15] but not others [16–20]. Collectively, convalescent plasma studies suggest that the variables that contribute to passive antibody efficacy in polyclonal preparations are not completely understood. Additionally, built on strong preclinical data showing the ability of antibodies to prevent infection [5], monoclonal antibody therapies have been developed, including combination products [21, 22]. Each of the three vaccines currently under emergency use in the United States induces neutralizing antibodies, often to levels exceeding those detected following natural infection [23–25].

However, whether elicited by vaccination or infection, antibody responses between individuals are highly variable [26–29], both in titer and in composition. This variability suggests that monoclonal antibody and convalescent plasma therapy, as well as vaccine design, can be improved by determining the factors that contribute to a functionally protective antibody response. Beyond neutralization, which has been established as a correlate of protection in diverse studies [22, 30–32], evidence has accrued supporting both protective and pathogenic roles of antibody effector functions in infection resistance and disease severity. These functions include activities mediated by both soluble factors and diverse innate immune effector cell types. For example, initiation of the complement cascade can result in direct viral or infected cell lysis [33], or modification of other activities including neutralization [33, 34]. Similarly, antibodies can induce phagocytosis, drive release of cytotoxic factors such as perforin and granzyme B, or secretion of inflammatory mediators such as a cytokines and reactive oxygen species [34, 35]. In studies of SARS-CoV-2, extra-neutralizing functions have been shown to play an important role in antiviral activity of antibodies [36–42]. The importance of these functions has been defined in vivo in animal models using both using Fc engineering to modulate binding of the Fc domain to Fcγ Receptors (FcγR), and through depletion of effector cells. In contrast, in correlative studies some extra-neutralizing functions have also been linked to disease severity [43, 44]. These findings suggest the importance of understanding the role of both neutralization and extra-neutralizing functions in antibody responses to SARS-CoV-2 infection. Given these observations, better understanding of the relationship between the magnitude and character of the humoral immune response and diverse antibody activities may offer key insights to further the development of successful therapeutics and vaccines for SARS-CoV-2.

## Results

### Characterization of antibody responses following SARS-CoV-2 infection

Antibody functions, including neutralization assessed by either an authentic virus assay or a luciferase-based pseudovirus assay, antibody-dependent cell-mediated phagocytosis (ADCP) mediated by monocytes, deposition of the complement cascade component C3b (ADCD), and FcγRIIIa ligation as a proxy for NK cell mediated antibody dependent cellular cytotoxicity (ADCC) induced by antibodies in response to recombinant antigen were previously reported [28] for a set of convalescent samples collected from a discovery cohort of 126 eligible convalescent plasma donors from the Baltimore/Washington D.C. area (Johns Hopkins Medical Institutions, JHMI) [29] and serum samples from 15 naïve controls and a validation cohort of 20 convalescent subjects from New Hampshire (Dartmouth-Hitchcock Medical Center, DHMC) [45] (Additional file 1: Table S1). Biophysical antibody features were defined by a customized multiplexed Fc array assay that characterizes both variable fragment (Fv) and Fc domain attributes across a panel of SARS-CoV-2 antigens, consisting of: nucleocapsid (N) protein, stabilized (S-2P) [46] and unstabilized trimeric spike protein, spike subdomains including S1 and S2, the receptor binding domain (RBD), and the fusion peptide (FP) from SARS-CoV-2; in addition, the panel included diverse pathogenic, zoonotic, and endemic coronavirus spike proteins and subdomains. Influenza hemagglutinin (HA) and herpes simplex virus glycoprotein E (gE) were evaluated as controls. The Fc domain characteristics evaluated for each antigen specificity included antibody isotype, subclass, and propensity to bind Fc receptors (FcRs) (Additional file 1: Table S2).

**Fig. 1:**
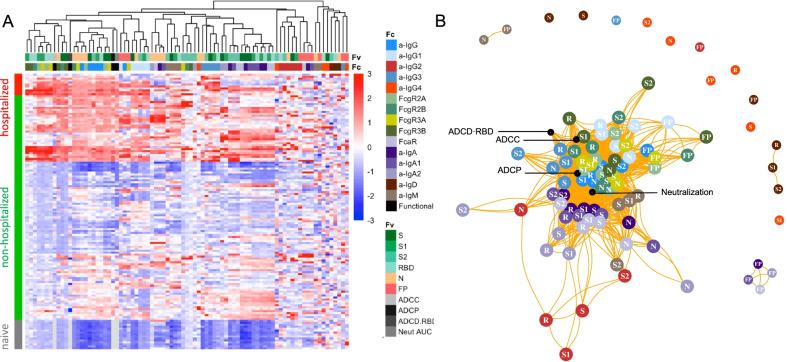
Biophysical and functional antibody responses among convalescent donors. **A**. Heatmap of filtered and hierarchically clustered SARS-CoV-2-specific Fc array features across disease severity and infection status in the JHMI cohort. Each row represents an individual subject, and subjects are grouped by disease status, as indicated by the vertical color bar. Each column represents an Fc array feature; horizontal color bars indicate each function or each Fv-specificity and Fc-characteristic tested. Responses are scaled and centered per feature and the range was truncated ± 3 SD. Higher responses are indicated in red and lower responses are indicated in blue. Missing data is indicated in light gray. **B**. Weighted network plots of correlative relationships (|r|> 0.5) among antibody functions (black) and CoV-2-specific antibody features. Fc array measurements are colored by Fc characteristic and Fv specificity is indicated in text label (R = RBD)

To understand how the different facets of the Ab response relate to one another, hierarchical clustering was performed on the biophysical antibody profiles of convalescent plasma donors (JHMI) and compared to the serum profiles of SARS-CoV-2 naïve subjects. Extensive variability in the SARS-CoV-2-specific Ab response magnitude and character was noted (Fig. 1A). High levels of IgG were observed in many individuals, particularly those who had been hospitalized, while a small number of convalescent donors appeared not to seroconvert despite documented infection via nucleic acid amplification. Similarly, there was considerable variability in the IgA and IgM responses in SARS-CoV-2-convalescent subjects. IgG2, IgG4, and IgD responses were less commonly observed. Distinctions in antibody responses between subjects were apparent among antigen specificities. For example, perhaps due its high homology with endemic CoV, FP responses were isotype switched consistent with an amnestic response, whereas IgM responses to S were reliably observed.

A weighted network plot depicting Pearson’s correlation coefficients between Fc array features and functional measurements was created to elucidate correlative relationships more directly between aspects of humoral responses (Fig. 1B). As was apparent in the heatmap (Fig. 1A), features were often more strongly grouped by Fc domain characteristics than antigen-specificity. Nodes representing antibody effector functions clustered more tightly with RBD-, S1-, S- and N-specific FcγR-binding levels, IgG3, and total IgG responses than with IgG1 responses or those directed at S2 or FP. Though most closely linked to IgG-associated features, neutralization potency appeared as a hub that connected to IgA and IgM responses. Based on both hierarchical clustering and correlation analysis (Fig. 1B), the ability of antigen-specific antibodies to interact with diverse FcγR was well correlated to multiple antibody effector functions.

### Multivariate modelling methods to predict functional responses

**Fig. 2:**
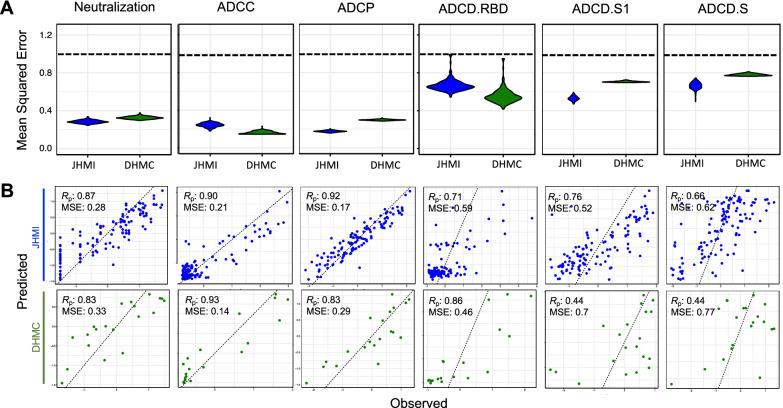
Multivariate linear regression modeling validation in test set. **A**. Comparison of mean-squared error between testing (JHMI) and validation (DHMC) data sets for each functional assay across cross-validation replicates. Dotted line indicates median performance on permuted data in the setting of repeated cross-validation. **B**. Correlation between predicted and observed responses in the discovery (JHMI, blue) and validation (DHMC, green) cohorts. Pearson correlation (Rp) and mean squared error (MSE) are reported in inset. Dotted line indicates x = y

With the dual goals of better understanding the humoral response features that may drive complex antibody functions and enabling robust predictions from surrogate measures, we applied supervised machine learning methods to this (JHMI) dataset, while using the DHMC cohort as validation to determine whether the models could predict activity in a generalized manner. A regularized generalized linear modeling approach trained to utilize Fc Array features to predict each antibody function with minimal mean squared error was selected based on prior success in identifying interpretable factors that contribute to functional activity while avoiding overfitting [47]. Five-fold cross-validation was employed to evaluate generalizability within the JHMI cohort, and comparison to models trained on permuted functional data established model robustness (Fig. 2A). The cross-validated models trained on diverse data subsets showed similar accuracy (measured by mean squared error) when applied to held out subjects as when used to predict effector function and neutralization activity that was observed in the validation cohort (DHMC). Model quality was also evaluated in terms of the degree of correlation between predicted and observed activity for a representative cross-validation replicate, allowing for better visualization of model performance (Fig. 2B).

**Fig. 3:**
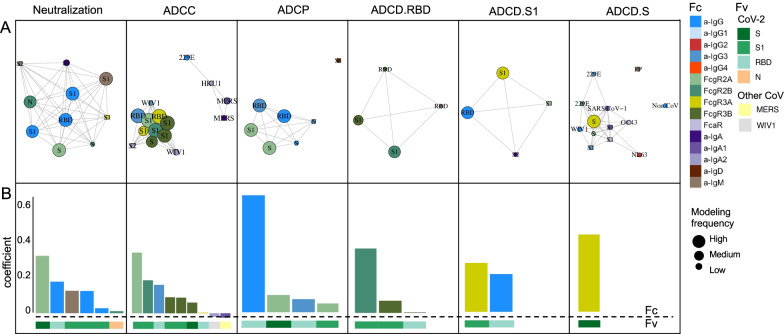
Final predicted biophysical features and contributions in the multivariate linear regression modeling. **A** Network showing the identity, relative degree of correlation, and frequency with which features contribute to models in the setting of repeated cross-validation. **B** Coefficients of biophysical features to the final models predictive of each function. Antigen specificity (Fv) and Fc characteristics (Fc) are shown in color bars

The model consistently selected a subset of features for each function (Fig. 3A). The features that appeared with high frequency in repeated modeling were likely to have relatively high coefficients, and inversely, biophysical features with relatively small coefficients were prone to be influenced by the selected sample subset and to be removed by chance across the replicates. Collectively, the frequently contributing features were exclusively related to spike recognition and were primarily driven by IgG and FcγR-binding antibodies.

To evaluate the magnitudes of feature contributions, a representative model for each function demonstrating the identity and relative coefficients of the contributing features is presented (Fig. 3B). Again, despite their sparseness compared to the control antigen, endemic CoV, and other epidemic CoV features, these models relied almost exclusively on antibody responses to the SARS-CoV-2 spike. Consistent with the experimental approach evaluating functions elicited specifically against RBD, ADCC and ADCP models depended principally on antibodies specific to RBD or more broadly to S1. In contrast, the lead feature for virus neutralization was recognition of stabilized spike (S-2P). Similarly, complement deposition against whole spike was best predicted by a single feature related to spike trimer recognition. Responses to the S2 domain were not observed to contribute to functional predictions. Intriguingly, IgA responses against other CoV were observed to make inverse contributions to ADCC predictions. While these contributions were of small magnitude, this result suggests the possibility that cross-reactive, potentially S2-specific IgAs may inhibit the activity of S-reactive IgGs, as has been observed in the context of the HIV envelope glycoprotein [48].

Beyond specificity, distinct antibody Fc characteristics contributed to model predictions. The most frequent Fc characteristic of features contributing to the final model of neutralization potency was the magnitude of IgG response, consistent with neutralization being FcR-independent. In contrast, the most frequent Fc characteristics in modeling ADCC and ADCP were FcγRIII- and FcγRII-binding responses, respectively—the receptors most relevant to each function. Further, despite comprising a relatively small fraction of circulating IgG, but consistent with its enhanced ability to drive effector functions [49, 50], IgG3 antibodies specific to RBD made a substantial contribution to models of both ADCP and ADCC activity, suggesting the potential importance of this subclass.

**Fig. 4:**
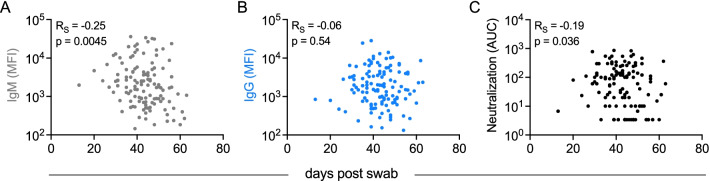
Relationships between RBD-specific IgM, IgG, and neutralization and days post swab. **A–C**. Scatterplots of RBD-specific IgM (**A**), IgG (**B**), and neutralization AUC (**C**) versus days post swab. Spearman correlation coefficients (R_S_) and p values are reported in inset

Intriguingly, S1-specific IgM contributed to models of neutralization potency. IgM is typically associated with initial exposures [51], suggesting the possibility that this feature represents de novo rather than recalled cross-reactive lineages that may exhibit superior neutralization activity across diverse isotypes, as has been observed in the context of influenza responses [52, 53]. Alternatively, the contribution of IgM to neutralization models may be directly mechanistic. Because the induction kinetics and persistence of differing Ig isotypes and neutralizing antibody activity are known to differ, we began to investigate the potential biological relevance of IgM responses to neutralization by evaluating how IgM, IgG, and neutralization activity varied in association with time since infection (Fig. 4). Whereas the magnitude of IgG responses to RBD did not show a statistically significant association with time in this cross-sectional analysis (Fig. 4B), both RBD-specific IgM and neutralization activity were inversely correlated with time since infection (Fig. 4A, C), suggesting that elevated IgM levels may contribute to elevated neutralization activity, particularly at timepoints early in the course of infection.

Overall, while functions were predicted with differing degrees of accuracy, each generalized well to the independent validation cohort and relied upon features with established biological relevance.

### Experimental validation of predictive models of antibody function

**Fig. 5:**
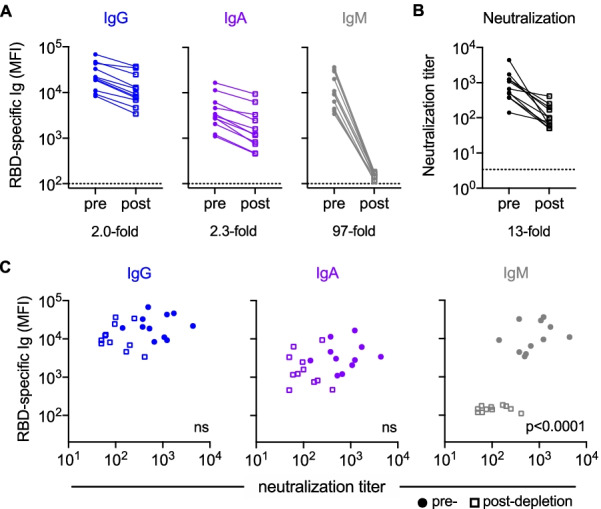
Experimental validation of IgM-mediated neutralization. **A**. Median fluorescent intensity (MFI) levels of RBD-specific IgG, IgA, IgM observed pre- (filled circles) and post- (hollow squares) IgM depletion. Mean fold change in MFI across samples for each isotype is indicated below the figure. **B**. Neutralization titers pre- and post-IgM depletion. **C**. Comparison of RBD-specific Ig levels to neutralization titer. Statistical significance (two-tailed p value) of Spearman correlation coefficients reported in inset

Given the somewhat surprising appearance of an IgM feature in predictions of neutralization activity, we sought to evaluate the mechanistic relevance of this isotype in particular. In a select group of individuals with both high IgM and neutralization levels (n = 11), IgM was depleted from serum to determine whether the loss of CoV-2-specific IgM resulted in a reduction in neutralization of SARS-CoV-2 (Additional file 1: Figure S1). Both total (not shown) and RBD-specific IgM was depleted (97-fold) (Fig. 5A). Minimal effects on total (not shown) and RBD-specific IgG (2.0-fold) and IgA (2.3-fold) levels were observed in the IgM-depleted samples. Following IgM depletion, samples showed 1.6- to 73-fold decreases in neutralization titer (Fig. 5B). Though the magnitude of changes in Ig levels and neutralization before and after depletion varied per donor, only IgM and not IgG or IgA levels showed a statistically significant correlation with neutralization titer in these individuals (Fig. 5C). This result demonstrates that mechanistically relevant features can be discovered from unbiased data analysis and modeling processes.

## Discussion

SARS-CoV-2-specific antibodies can drive varied antiviral functions beyond neutralization [36, 45, 54]. These responses have been less well characterized, but accumulating evidence suggests their importance to protection from infection and disease. Both ADCC and phagocytosis have been reported to contribute to antibody-mediated antiviral activity against other coronaviruses [55–57]. Collectively, these functions have been suggested to play an important role in defense against SARS-CoV-2; they have been implicated in in vivo protection in diverse studies, including passive transfer studies that have demonstrated that effector functions play a role in the antiviral activity of monoclonal antibodies and correlates of protection analysis carried out on vaccine candidates [36–42]. Fc engineering approaches that both knocked out or enhanced antibody effector functions and studies of the depletion of effector cells in the context of diverse antibodies have provided convincing evidence of the mechanistic relevance of these observations.

In this work, antibody functions measured in two cohorts of convalescent subjects were modeled using biophysical antibody profiles comprised of tandem attributes representing Fv- specificity and Fc characteristics. Multivariate linear regression identified distinct biophysical features that predicted antibody functions such as ADCC, ADCP, ADCD, and neutralization, showing the unique dependencies of each activity on different aspects of humoral responses. Although responses toward both endemic and pathogenic CoV were considered, models were almost exclusively reliant on SARS-CoV-2-specific responses in predicting functional activity. These predictions were robust and generalizable, performing similarly well in training and testing data subsets across cross-validation runs as in an independent validation cohort. The consistency between antibody features contributing to each modeled function and expected biological relevance suggests that modeling approaches such as that employed here can identify mechanisms of antibody activity that might apply across diverse vaccine regimens and plasma and monoclonal antibody therapy. Agreement across vaccine regimens and between preclinical models and clinical studies have been observed previously [58–60].

Spike-specific FcγR-binding antibodies made frequent contributions to models of effector functions, with FcγRIIa contributing strongly to phagocytosis and FcγRIIIa contributing strongly to NK cell activity. Among subclasses, IgG3 made an outsized contribution, consistent with prior studies in the context of other infections [61–63], and monoclonal antibody subclass-switching studies [49, 50]. In contrast, virus-specific IgM contributed to predictions of neutralization activity. The relationship between IgM responses, neutralization potency, and time seen here is consistent with prior reports [64]. SARS-CoV-2 specific IgM has attracted interest because of its association with lower risk of death from COVID-19 [26]. Consistent with our experimental results, another study in which IgM was selectively depleted also observed resulting reduction in neutralization activity, but additionally confirmed the activity of the isolated IgM fraction [65, 66]. Interestingly, SARS-CoV-2 specific IgM administered intranasally has been shown to be effective in treating novel SARS-CoV-2 variants of concern, including the alpha, beta, and gamma variants in a mouse model [67]. The finding that so much of the neutralizing activity of convalescent plasma against SARS-CoV-2 resides in the IgM fraction raises concern about that gamma globulin preparations may lose much of their antiviral activity as this isotype is removed. Similarly, the faster clearance profile of IgM as compared to IgG may hold implications for both frequency of dosing and timing of plasma donation.

While features contributing to functional predictions have both prior support from other studies and experimental validation within this cohort, other feature sets are likely to provide similar performance. Given high feature dimensionality and relatively fewer subjects, regularization was used to increase the quality of prediction. This approach simplified the resulting models, resulting in improved interpretability of the selected variables at the cost of eliminating features that are highly correlated to selected variables in the established model. Collectively, this modeling choice can result in a trade-off between model simplification and obscuring potential biological mechanisms. Other limitations include the use of surrogate functional assays that bear advantages in terms of throughput and reproducibility but pose limitations in terms of their biological relevance. As further functional assays reliant on free virions and infected cells are developed, it will be of interest to compare and contrast both the degree of correlation with these convenient proxy assays as well as to model those activities in pursuit of insights into unique subpopulations of antibodies that may be responsible for their induction, or to define general characteristics of a response that is highly polyfunctional.

Insights into the features of functionally potent antibodies raised in the context of vaccination and across viral variants of concern are of additional interest. While good predictive performance might be anticipated given the consistency of these models with expectations based on basic antibody biology, it is possible that the overall profiles of antibodies induced by vaccination, or by different vaccines, could be sufficiently substantial as to disrupt correlative relationships that are not mechanistically rooted. To this end, while robust relationships between virus-specific IgM and neutralization have been reported in several studies of vaccine recipients [68–71], the direct ability of these models to predict the activity of vaccine-induced responses remains to be determined.

## Conclusions

As viral variants continue to emerge, rapid binding profiling may be an important complement to functional breadth assessments. Insights into how Fc characteristics of cross-reactive responses relate to diverse functions may provide accelerated insights into population-level susceptibility and support prioritization among candidate vaccine regimens. Numerous randomized clinical trials of convalescent plasma for COVID-19 are in the process of completion and it is likely that plasma remnants will be available for retrospective detailed serological analysis and correlation with clinical outcome [16]. This multivariate analysis provides a blueprint for carrying out such investigation, which could provide information on the antibody functions that contribute to clinical efficacy. The discovery of antibody functions associated with passive antibody efficacy could allow optimization of serological characteristics of mAbs, plasma and gamma globulin products for prevention and therapy of COVID-19.

## Methods

### Human subjects

The discovery cohort comprised 126 adult eligible convalescent plasma donors diagnosed with SARS-CoV-2 infection by nucleic acid amplification in the Baltimore, MD and Washington DC area (Johns Hopkins Medical Institutions, JHMI cohort) and has been previously described [29]. The validation cohort comprised 20 SARS-CoV-2 convalescent individuals from the Hanover, New Hampshire area (Dartmouth Hitchcock Medical Center, DHMC cohort) [45]. This cohort was selected on the basis of availability and collection at a similar timepoint in the pandemic, offering rigor by virtue of somewhat differing demographics between New Hampshire and Maryland (greater disease severity and age in the DHMC cohort), differing sample collection (serum versus plasma), and differing handling and testing (neutralization assays used) processes. Infection with SARS-CoV-2 was confirmed in all convalescent subjects by nasopharyngeal swab PCR. Plasma (JHMI) or serum (DHMC) was collected from each donor approximately one month after symptom onset or first positive PCR test in the case of mild or asymptomatic disease. Samples from 15 naïve subjects collected from the New Hampshire area early in the pandemic served as negative controls. Seronegative status among these presumed naïve subjects was subsequently confirmed by clinical laboratory assays. Additional file 1: Table S1 provides basic clinical and demographic information for each cohort.

Human subject research was approved by both the Johns Hopkins University School of Medicine’s Institutional Review Board and the Dartmouth-Hitchcock Medical Center Committee for the Protection of Human Subjects. All participants provided written informed consent.

### Antibody features and functions

The magnitude, Fv specificity, and Fc domain characteristics of antibody responses to diverse coronavirus and control antigens were profiled by multiplexed Fc Array assay [22], as previously described [28, 45, 72]. Additional file 1: Table S2 reports the complete list of antigen specificities and Fc domain characteristics that were assayed. Fc Array data reported in median fluorescent intensity (MFI) was log transformed prior to analysis.

Antibody functions were assayed as previously described [28, 45]. Briefly, neutralization of authentic virus [29, 73, 74] was determined for samples from the JHMI cohort, whereas a pseudovirus neutralization assay [75] was employed for evaluation of the DHMC cohort. Phagocytic activity was defined as the level of uptake of antigen-conjugated beads by THP-1 monocytes (ADCP) [76, 77] or primary neutrophils (ADNP) [78]. ADCC activity was modeled using a reporter cell line that expresses luciferase in response to FcγRIIIa ligation [79]. Antibody-dependent complement deposition was assessed by measuring C3b levels on antigen-conjugated beads following incubation in complement serum [80]. For each assay, SARS-CoV-2 naïve samples were employed as negative controls, and data was collected in replicate.

IgM depletion.

IgM was depleted from serum as described previously [65]. Briefly, 200 μL of NHS HP SpinTrap resin (Cytiva) was equilibrated and used to immobilize anti-human IgM (μ-chain specific, Sigma I0759) at 850 μg/mL for 30 min with end-over-end mixing at room temperature. The resin was washed, quenched with 50 mM Tris HCl, 1 M NaCl pH 8.0 and 0.1 M sodium acetate 0.5 M NaCl pH 4, and incubated with serum diluted 1:5 in DMEM and incubated overnight at 4 °C with end-over-end mixing. Flow-through was subsequently collected by centrifugation. IgG, IgA, and IgM levels of each selected sample were evaluated with and without IgM depletion by multiplex assay as described above [28, 45, 81]. Neutralization was measured by pseudovirus reporter assay as described above [75].

### Data analysis and visualization

Basic analysis and visualization were performed using GraphPad Prism. Heatmaps, correlation plots, and other graphs were generated in R (supported by R packages pheatmap [82], igraph [83], and ggplot2 [84]). Fc Array features were filtered by elimination of features for which the samples exhibited signal within 10 standard deviations (SD) of the technical blank. Log transformed SARS-CoV-2-related Fc Array features and selected functions were scaled and centered by their standard deviation from the mean (z-score) per cohort and visualized following hierarchical clustering according to Manhattan distance. A weighted correlation network of pairs of SARS-CoV-2-related features and selected functions for which Pearson’s correlation coefficient ≥ 0.5 was graphed.

Multivariate linear regression was employed to predict antibody functions based on biophysical features with the R package “Glmnet” [85], as previously described [58–60]. Regularization by L1-penalization (LASSO) was applied to eliminate variables that were less relevant to the outcome by imposing a penalty on the absolute value of the feature coefficient in order to reduce overfitting and reinforce performance generalizibility^86^. Functional measurements of ADCP, neutralization, and S1-specific ADCD were log_10_ transformed to reduce the prediction error of the models based on the assumption that better fitting models were more likely to rely on biologically relevant features. The lambda parameter (λ) was tuned using five-fold cross-validation to minimize mean squared error (MSE). A process of 200-times repeated modeling was used to investigate the potential of the different combinations of the biophysical features for modeling. Established with the JHMI cohort, a final model was selected based on the median MSE obtained among the repeated run in the JHMI cohort. The selected features and their coefficients were reported at a value of λ at which median model performance fell one standard error above the minimum to optimize the generalizability and provide more regularization to the model. In the permutation test procedure, the penalized multivariate regression was performed against randomized functional outcomes in the JHMI cohort in a 200-time repeated fashion. The correlation network was conducted with the biophysical features that were repeatedly selected within the repeated modeling process.

## Supplementary Information

**Additional file 1: Table S1.** The expression of 1879 IRGs in GSE98793 dataset.

## Data Availability

The datasets used and analyzed are available at: https://github.com/AckermanLab/Butler_et_al_COVID_2020. https://github.com/AckermanLab/Natarajan_et_al_COVID_2021

## Abbreviations

ACDP: Antibody-dependent cellular phagocytosis
ADCC: Antibody-dependent cellular cytotoxicity
ADCD: Antibody-dependent complement deposition
COVID-19: Coronivirus 2019 disease
DHMC: Dartmouth Hitchcock Medical Center
Fc: Fragment crystallizable
FcγR: Fc gamma receptor
FcR: Fc receptor
FP: Fusion peptide
Fv: Fragment variable
gE: Herpes simplex virus glycoprotein E
HA: Influenza hemagglutinin
IgA: Immunoglobulin A
IgG: Immunoglobulin G
IgM: Immunoglobulin M
JHMI: Johns Hopkins Medical Institutions
MERS: Middle Eastern Respiratory Syndrome
MSE: Mean squared error
N: Nucleocapsid
RBD: Receptor binding domain
S-2P: Stabilized spike
SARS-CoV-2: Severe Acute Respiratory Syndrome Coronavirus 2
SD: Standard deviation
