# Supplementary material for: Antibody attributes that predict the neutralization and effector function of polyclonal responses to SARS-CoV-2
Source: BMC Immunol. 2022 Feb 16;23:7. doi: 10.1186/s12865-022-00480-w (PMC8851712; doi:10.1186/s12865-022-00480-w)
Supplement: Supplementary file 1 — Additional file 1: Table S1. The expression of 1879 IRGs in GSE98793 dataset. [file 12865_2022_480_MOESM1_ESM.pdf]

Supplemental Materials

Supplemental Figure	
Supplementary Figure 1	Samples selected for IgM depletion
**Supplemental Tables**	
Supplemental Table 1	Cohort characteristics
Supplemental Table 2	Fc detection and antigen reagents

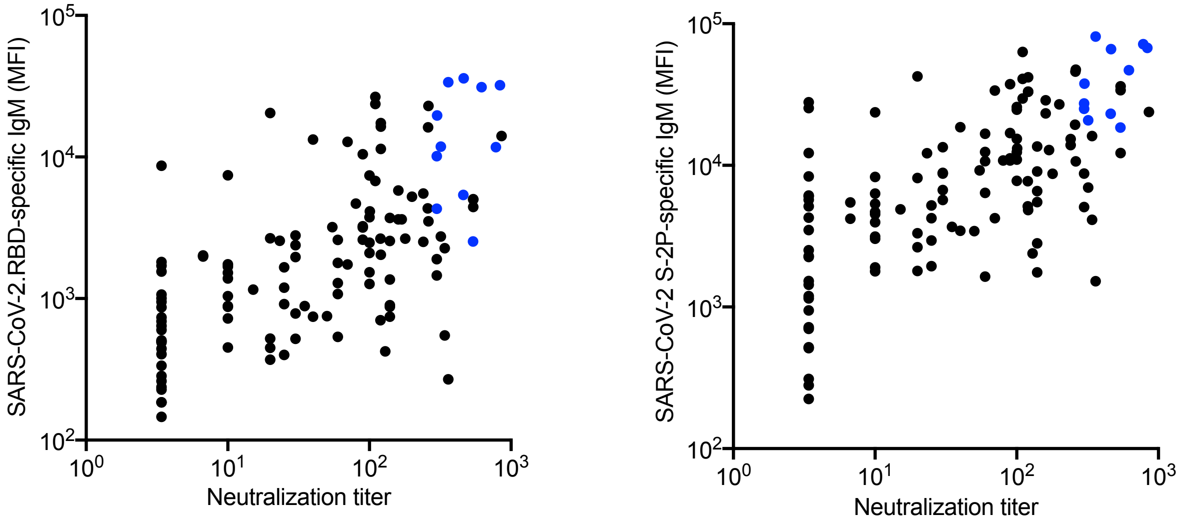

Supplemental Figure 1. Samples selected for IgM depletion. Comparison of SARS-CoV-2 RBD-specific (left) and stabilized spike (S-2P)-specific IgM levels (median fluorescent intensity, MFI) and neutralization titer. Samples with high IgM and high neutralization titers that were selected for depletion are highlighted in blue.

Supplemental Table 1. Cohort characteristics. Demographic information on convalescent serum (DHMC) and convalescent plasma samples (JHMI). NA indicates not applicable and IQR indicates interquartile range. Reproduced with modifications from Natarajan, et. al, 2021[26].

**Characteristic**	**JHMI Convalescent** n=126	**DHMC Convalescent** n=20	**Naive** n=15
Median age (IQR), years	42 (29-53)	54 (45-62)	34 (28-52)
Sex			
Female	58 (46%)	10 (50%)	8 (53.3%)
Male	68 (54%)	10 (50%)	7 (46.7%)
Hospitalized (severity)			
No	114 (90.5%)	16 (80%)	NA
Yes	12 (9.5%)	4 (20%)	NA
Median age (IQR), years	45 (42-51)	66.5 (63-70)	
Median days since PCR+ or symptom onset (IQR)	43 (38-48)	38 (33-45)	NA

Supplemental Table 2. Fc detection and antigen reagents

Antigen	Source	Fc Detection	Source
H1N1 HA1	Immune Technology IT-003-00110p	a- IgG	Southern Biotech 1030-09
HSV gE	Immune Technology IT-005-005p	a-IgG1	Southern Biotech 9054-09
SARS CoV-2 N	Immune Technology IT-002-033Ep	a-IgG2	Southern Biotech 9070-09
SARS CoV-2 FP	New England Peptide	a-IgG3	Southern Biotech 9210-09
SARS CoV-2 S1 (for Fc Array)	ACRO Biosystems S1N-C52H3-100ug	a-IgG4	Southern Biotech 9200-09
SARS CoV-2 S1 (for ADCD)	Sino Biological 40150-V08B1-20	a-IgA	Southern Biotech 2050-09
SARS CoV-2 RBD	BEI Resources NR-52366	a-IgA1	Southern Biotech 9130-09
SARS CoV-2 S2	Immune Technology IT-002-034p	a-IgA2	Southern Biotech 9140-09
SARS CoV-2 S-2P	Expressed in Expi 293	a-IgM	Southern Biotech 9020-09
WIV1 S-2P	Expressed in Expi 293	a-IgD	Southern Biotech 9030-09
MERS S-2P	Expressed in Expi 293	FcγR1	Duke Protein Production Facility
MERS S1	Sino Biological 40069-V08B1	FcγR2a	Boesch, et. al, 2014
HCoV OC43 S	Sino Biological 40607-V08B	FcγR2b	Boesch, et. al, 2014
OC43 S-2P	Expressed in HEK 293F	FcγR3a	Boesch, et. al, 2014
229E S1	Sino Biological 40605-V08H	FcγR3b	Boesch, et. al, 2014
229E S-2P	Expressed in Expi 293		
HKU1 S1	Sino Biological 40606-V08H		
HKU S-2P	Expressed in Expi 293		
NL63 S1	Sino Biological 40604-V08H		
